# Peer Review of “A Physical Activity Mobile Game for Hematopoietic Stem Cell Transplant Patients: App Design, Development, and Evaluation”

**DOI:** 10.2196/28649

**Published:** 2021-04-13

**Authors:** Michael C Robertson

**Affiliations:** 1 Department of Rehabilitation Sciences The University of Texas Medical Branch Galveston, TX United States

**Keywords:** cancer, mobile app, gamification, bone marrow transplant, alpha testing, physical activity

*This is a peer-review report submitted for the paper "A Physical Activity Mobile Game for Hematopoietic Stem Cell Transplant Patients: App Design, Development, and Evaluation."*

## Round 1 Review

### General Comments

I appreciate the chance to review this study [1], and I applaud the authors for their pursuit of an important topic. This paper details the development and usability testing of Walking Warrior, a mobile app designed to help increase physical activity (PA) levels in individuals who have undergone hematopoietic stem cell transplant (HSCT). The study presents some important and valuable insights into the development process of an mHealth (mobile health) app. I appreciate that there may exist some tension between more formative app development and the level of strict adherence to scientific principles that one would expect in late-stage efficacy testing, but nonetheless, I believe the manuscript as written is not yet sufficiently grounded in scientific frameworks, theory, models, or methods to be suitable for publication. I offer some suggestions below.

### Specific Comments

#### Major Comments

##### Introduction

1. I would recommend further developing the link between how increasing PA can positively impact HSCT patients; at present, this is not sufficiently developed.
2. The reference for the statement “Unfortunately, adherence to recommended levels of PA is low in cancer patients” is not appropriate.
3. The rationale and argument for the use of gamification and game design elements to increase physical activity in cancer survivors are not sufficiently developed. I recommend incorporating some of the relevant theory and literature detailing why this approach may be useful for physical activity promotion in this population.
4. Develop the gap in the literature—why is the lack of PA mobile apps specifically for HSCT patients important? What unique challenges faced by this population may make existing PA app options less than ideal?
5. The stated hypothesis, that the game will “motivate HSCT patients to walk,” does not appear to align with the study aims centered on expert heuristic usability evaluation.
6. Why would you hypothesize that the “game will motivate HSCT patients to walk due to…continued game play requires walking: if they want to play more, they will need to walk”? This is not readily apparent.

##### Methods

1. There does not appear to be a scientific model, framework, or theory undergirding the development process. I suggest taking care to align the development process with an existing scientific approach. You state that “The entire development process was based on user-centered design,” but there is no accompanying citation, and it is not clear what this entails.
2. It was stated that “A 40-item expert heuristic questionnaire was designed and validated,” but this does not seem to be the case. How was the instrument validated?
3. The qualitative data analysis methods do not seem to have been grounded in a scientific framework. A description of the hierarchical factor analysis methods is not present in the *Methods* section.

##### Results

1. I would recommend providing more information about the characteristics of the study sample.
2. Interpretation of the descriptive statistics seems arbitrary. Are there normative values that can be referenced?
3. Key methods are presented in the *Results* section (eg, “To improve the accuracy of our step counters of our designed WW, we recruited 5 additional usability evaluators who were nursing informatics graduate students”).

##### Discussion

1. The discussion should present study findings in the context of the existing literature. How do your results compare to other studies centered on usability testing of physical activity apps?

## Round 2 Review:

I thank the authors for their responsiveness to reviewer comments. I do have remaining concerns that need to be addressed before this manuscript is suitable for publication.

1. At the end of the *Introduction* section, you state:
  "We hypothesize that our game will motivate HSCT patients to walk due to: (1) large portion of HSCT patients earlier reported to enjoy playing match-3 puzzle game such as Candy Crush which is similar to our game; (2) continued game play requires walking: if they want to play more, they will need to walk; (3) patients are educated that walking is part of their therapy, playing the game reinforces this behavior; 4) walking will allow players to unlock additional levels and allows them to earn higher scores; (5) game playing and walking performance data are automatically collected and displayed on a website that allows patient self-tracking and provider review; (6) the game is mentally challenging, this provides entertainment, logical thinking opportunity, the element of chance, and high replayability; (7) tiles to move in the puzzle are displayed as cell types and medications which are relevant to the HSTC patients’ condition and provide education to players enhancing their knowledge of the underlying biology and treatment they receive; (8) in addition to their automatically collected data, patients will participate in a survey which will serve as a tool for software evaluation and additional development showing the individual patient’s true experience and opinions are valued and integrated into the next phase of software development.
  However, the purpose of this project is not to test these or any hypotheses. Please revise accordingly, removing all references to hypotheses (which imply that they will be tested).
2. You state that “A 40-item expert heuristic questionnaire was designed and validated.” I appreciate that you provided more details in response to previous comments. However, based on what you have shared, I believe that claiming to have “validated” the questionnaire would be misleading. Please revise. For example, you may remove the word “validated” and say something like, “Two experts assessed the face validity of the 40-item expert heuristic questionnaire.” This is a subtle but important distinction.
3. Additionally, building on comment #2, the fact that this was not a measure with established psychometric properties is a limitation of the study that needs to be explicitly addressed as a limitation in the *Discussion* section.
4. I am skeptical of the finding articulated in the abstract as “Findings from the expert usability evaluation suggest the game’s assets of clarity, ease of use, appropriateness, quality, walking motivation, and mental effort were all favorable.” In the *Results* section, you state, “although 2 categories’ means were close to neutral (3.1), which is considered favorable due to the wording of those items,” but taking a look at the actual items, this is not clear to me. Please provide more evidence or commentary to substantiate this claim, or otherwise revise accordingly. I think it could be useful to talk about some of the potential opportunities for improvement of this very interesting intervention.
5. Please provide evidence to support the claim that “HSCT patients…carry a smartphone.”
6. Consider revising the sentence, “There is no personally identifiable information in the database, only user’s names and performance data” to state “usernames,” not “user’s name,” if appropriate.
7. Please address the fact that there was only 1 bone marrow transplant nurse to complete the expert heuristic usability evaluation of WW as a limitation of this study in the *Discussion* section. This seems to be a major limitation, and that person’s scores seemed to be markedly different from the programmers’ scores in some domains. Please provide some commentary on this.
8. Related to this, this statement is quite unclear to me given that there was only 1 bone marrow transplant nurse: “Hierarchical cluster analysis confirmed that the bone marrow transplant nurse and the computer programmer neither least nor most represented their domain group.” Please clarify.
9. Please move this statement, “The process of using “game design elements in non-game contexts” is known as gamification [24]” to the *Introduction* section.

## Abbreviations

HSCT: hematopoietic stem cell transplant
mHealth: mobile health
PA: physical activity
